# MLS Laser Reduce Pain in Patients with Chronic Low Back Pain

**DOI:** 10.5812/aapm-158778

**Published:** 2025-02-25

**Authors:** Sara Arefi, Seyed Reza Saidian, Mohamadreza Mokhtari, Daryoush Eliaspour

**Affiliations:** 1 Ahvaz Jundishapur University of Medical Sciences, Ahvaz, Iran; 2 Shahid Beheshti University of Medical Sciences, Tehran, Iran

**Keywords:** MLS Laser, Chronic Low Back Pain, Treatment

## Abstract

**Background:**

The Multiwave Locked System (MLS) has been shown to reduce inflammation and enhance biostimulation.

**Objectives:**

The aim was to investigate the effect of using MLS laser in reducing pain severity in patients with chronic back pain.

**Methods:**

This randomized double-blind study was conducted on 30 patients (15 in each group) with chronic low back pain (LBP) who were referred to Imam Khomeini Hospital and diagnosed by a physical medicine specialist based on diagnostic criteria. The intervention group was treated with MLS multiple wave laser, performed 12 times, with patients receiving laser treatment twice a week. The control group was treated with exercise therapy. Pain intensity was evaluated using the Visual Analog Scale (VAS) before treatment and 6 weeks after treatment. Pain was analyzed before and after the intervention, both within and between groups.

**Results:**

Of the 30 evaluated patients, the mean and standard deviation of age (P = 0.392) and gender (P = 0.666) were not statistically significant between the two groups. The VAS value before treatment in the intervention and control groups was 7.66 ± 1.11 and 7.73 ± 1.16, respectively (P = 0.794). After treatment, the VAS values in the MLS and control groups were 5.60 ± 1.35 and 7.73 ± 1.16, respectively. Statistical evaluation showed a statistically significant difference between the two groups (P = 0.001), and the total change in VAS values was significant (P = 0.001).

**Conclusions:**

Multiwave Locked System laser can reduce the pain severity of chronic LBP, with the reduction rate in the intervention group being significantly higher than in the exercise group.

## 1. Background

Musculoskeletal pain is a worldwide disorder that transcends demographics and age. These disorders not only impact older individuals but also affect people across the age spectrum (1). Musculoskeletal disorders are the second leading cause of disability worldwide, with persistent pain largely attributed to musculoskeletal conditions (1, 2). During their lifetime, 60% to 80% of the population experiences low back pain (LBP), a form of musculoskeletal pain. Of these individuals with acute LBP, more than 30% may develop chronic LBP (3). Low back pain is a major health disorder with significant social and economic costs (4). It affects a large proportion of the population, and its toll on patients, society, and families makes improving treatment for this common, yet benign disorder a principal aim (5, 6). Low-level light therapy (LLLT) is an alternative approach to pharmacological management for chronic LBP (7). Despite its extensive use, the effectiveness of LLLT is controversial (8). Traditional methods include physical therapy, medication, education, and back exercises, but these methods do not help in all cases. A large number of patients seek alternative methods, such as LLLT (9, 10). The Multiwave Locked System (MLS), a type of LLLT laser, has been shown to reduce inflammation and enhance biostimulation, influencing tendons, enhancing the functionality of ligaments by reducing thickness, reducing the severity of patient pain, and increasing the function of myoblasts to enhance muscle tissue recovery (11). Additionally, MLS Laser has shown clinical improvement in vascular conditions, including Raynaud’s phenomenon (12). The MLS is used by various medical practitioners in Europe and the United States to decrease inflammation and pain in different cases (7). Many patients have experienced success with MLS treatments, including in orthopedic cases.

## 2. Objectives

The present study aimed to investigate the effect of using MLS laser in reducing pain severity in patients with chronic back pain.

## 3. Methods

### 3.1. Study Setting and Population

This randomized and double-blind study was conducted in a group of 30 patients with non-mechanical back pain who were referred to the Imam Khomeini Hospital clinic. These patients were diagnosed by a physical medicine specialist based on diagnostic criteria, which included pain in the low back or one or both lower extremities on most days for at least 3 months. The patients were randomly divided into two groups. After explaining the study plan to the patients and obtaining informed consent, they were divided into two groups of 15 each — control and intervention — based on a random list prepared in advance by a statistical consultant, taking into account the inclusion and exclusion criteria. The intervention group was treated with the MLS laser, while the control group received exercise therapy.

### 3.2. Inclusion and Exclusion Criteria

Patients with LBP, aged 18 to 65 years, and a pain duration of more than 3 months, with or without referred pain, were included in the study. Additionally, patients were required to have the ability to read and write to complete the questionnaire and provide written informed consent. Exclusion criteria included mechanical LBP, simultaneous involvement of nerve roots, neurological and sensory disorders, spondylolisthesis, severe instability of vertebrae, history of surgery in the lower back, severe osteoporosis, spinal cord infection, and lack of patient satisfaction. Patients with chronic diseases, including those with pacemakers, pregnancy, stimulators, and cancer, were also excluded.

### 3.3. Measurements

Based on the above criteria, samples were selected from referring patients by a specialist doctor. Patient information, such as age, gender, severity of back pain, history of back pain, and amount of back movement (degree), was recorded in the patient file. Patients completed the modified Oswestry Questionnaire and the consent form to participate in the study. After registering the basic information of the patients, they were divided into two treatment groups — exercise therapy and laser therapy — by the random replacement method with six blocks. Two types of exercise therapy protocols were chosen. The first protocol consisted of common exercise movements whose effectiveness was agreed upon and recommended to patients in treatment systems. The second protocol included sports movements that did not have any effect on the treatment of back pain and were essentially outside the back area. This protocol was performed as a placebo in the laser group only. The desired movements were taught to the patients, and their performance was monitored during the treatment. Laser therapy was performed with the MLS laser at eight points on the sides of seals 12 to 15 for 12 sessions, twice a week. Laser features included a 905 nm wavelength (25W/75W peak power). During the treatment, the course of the disease was noted in each patient file, and at the beginning and end of the treatment period, in the twelfth week, six weeks after the end of the treatment, information related to disability indicators, back movements, pain intensity, and patient satisfaction with the treatment process was recorded by a questionnaire. If the patients did not return, they were contacted to determine the reasons for not completing the treatment course. In this study, the Visual Analog Scale (VAS) method was used to measure the patients' pain, which includes a red horizontal strip with a length of 10 cm, where the patient indicates their pain status on the axis from zero to maximum.

### 3.4. Ethical Considerations

The study was conducted under the research committee and approved by the Research Ethics Board of Ahvaz University of Medical Sciences. The studies complied with the rules of the Helsinki Declaration and were approved with the ethical code IR.AJUMS.HGOLESTAN.REC.1401.115 and the IRCT code IRCT20230827059275N1.

### 3.5. Statistical Analysis

SPSS statistical software version 26 was used for data analysis. Numerical data are presented as mean and standard deviation, while qualitative data are displayed as frequency. For comparison, a one-way analysis of variance (ANOVA) and the post hoc Tukey method were used to compare groups, and the chi-square test was used to compare qualitative values. The level of significance in this study was set at 0.05.

## 4. Results

**Figure 1.**
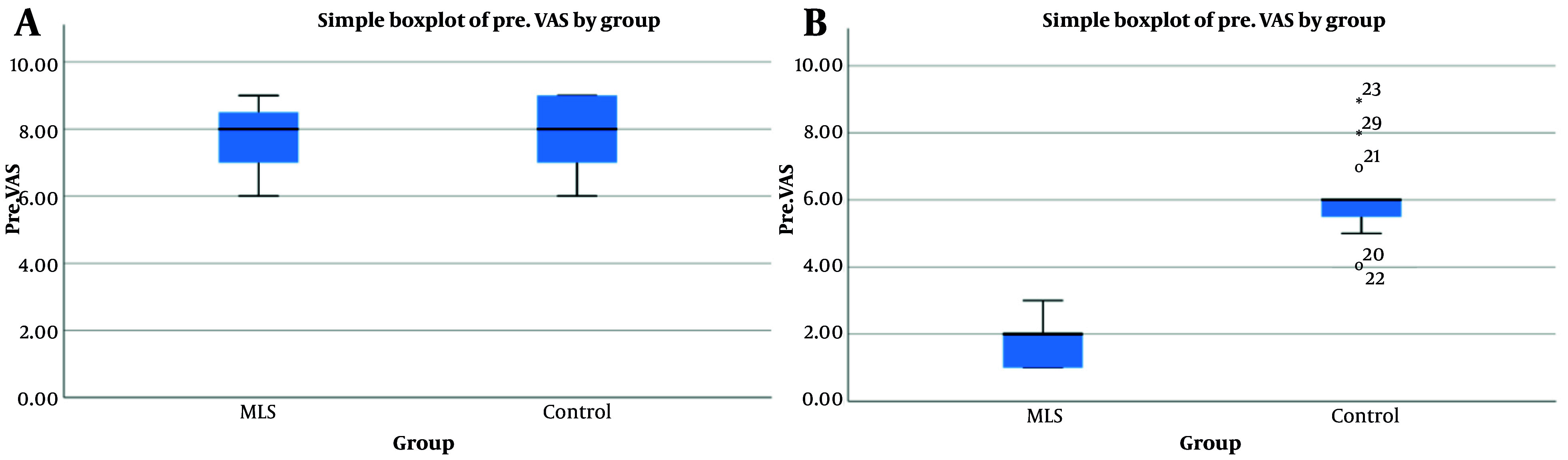
Evaluation of Visual Analog Scale (VAS) Index in two groups before and after treatment

Of the 30 evaluated cases, the mean and standard deviation of age in the intervention group was 47.73 ± 4.93 years, and in the control group, it was 49.13 ± 3.83 years. Based on the statistical evaluation, the two groups did not have a statistically significant difference (P = 0.392). Additionally, a total of 23 cases were male and 7 cases were female, and there was no statistically significant difference between the two groups (P = 0.666) (Table 1). The mean and standard deviation of the VAS before treatment in the intervention and control groups were 7.66 ± 1.11 and 7.73 ± 1.16, respectively, and there was no statistically significant difference between the two groups (P = 0.794). However, their values after treatment in the MLS and control groups were 7.73 ± 1.16 and 5.60 ± 1.35, respectively. Based on the statistical evaluation, there was a statistically significant difference between the two groups (P = 0.001), indicating that pain severity was lower in the intervention group (Figure 1). Additionally, by comparing these values before and after treatment in the two groups with a paired *t*-test, the difference in VAS was significant (P = 0.001), with greater pain reduction in the intervention group (Table 2). By evaluating the Pin Prick Index, the ratio of normal to abnormal before treatment was 8/7 in the intervention group and 9/6 in the control group. This index after treatment was 13/2 in the intervention group and 10/5 in the control group, and based on statistical evaluation, the two groups had a significant difference (P = 0.046). Regarding the Light Touch Index, the ratio of normal to abnormal before treatment was 8/7 in the intervention group and 7/8 in the control group. This index after treatment was 14/1 in the intervention group and 7/8 in the control group, and based on statistical evaluation, the two groups had a significant difference (P = 0.001) (Table 3). The recovery rate of EMG.NCS in the MLS laser group was significantly higher than in the control group, and this difference was significant based on statistical evaluation in the two groups (P = 0.001) (Table 4). 

**Table 1.** Comparison of Demographic Information of Patients

Variables	Groups		P-Value
	Intervention	Control	
**Age (mean ± SD)**	47.73 ± 4.93	49.13 ± 3.83	0.392
**Gender (M/F)**	12/3 (80/20)	11/4 (73.3/26.7)	0.666

**Table 2.** Evaluation of Visual Analog Scale Index in Two Groups Before and After Treatment ^a^

Variables	Group		P-Value
	Intervention	Control	
**VAS**			0.001
Pre-intervention	7.66 ± 1.11	7.73 ± 1.16	0.794
Post-intervention	1.86 ± 0.74	5.60 ± 1.35	0.001

Abbreviation: VAS, Visual Analog Scale.

^a^ Values are expressed as mean ± SD.

**Table 3.** Evaluation of Pin Prick Index in Two Groups Before and After Treatment

Variables (Normal/Abnormal)	Groups		P-Value
	Intervention	Control	
**Pin Prick**			0.046
Pre-intervention	8/7 (53.3/46.7)	9/6 (60/40)	0.713
Post-intervention	13/2 (86.7/13.3)	10/5 (66.7/33.3)	0.195
**Light touch**			0.001
Pre-intervention	8/7 (53.3/46.7)	7/8 (46.7/53.3)	0.715
Post-intervention	14/1 (93.3/6.7)	8/7 (53.3/46.7)	0.013

**Table 4.** Evaluation of EMG.NCS Index in Two Groups Before and After Treatment ^a^

Variables	Groups		P-Value
	Intervention	Control	
**EMG.NCS**			0.001
Pre-intervention			0.666
Normal	0 (0)	0 (0)	
L5-S1 involvement	4 (26.6)	3 (20)	
L4-L5 involvement	11 (73.4)	12 (80)	
Post-intervention			0.0001
Normal	13 (86.7)	2 (13.3)	
L5-S1 involvement	0 (0)	2 (13.3)	
L4-L5 involvement	2 (13.3)	11 (73.4)	

^a^ Values are expressed as No. (%).

## 5. Discussion

In the present study, the effectiveness of MLS laser therapy in treating chronic LBP was assessed, and it was found that the laser resulted in greater pain reduction and functional improvement compared to exercise. Similarly, Mehrdad et al. reported that the combination of exercise and MLS laser therapy led to the most significant improvement in chronic LBP (13); however, our study did not investigate the combination of these two methods.

A study by Akbari et al. evaluated the effect of low-power laser therapy on neck pain and found that pain levels in the muscle energy group decreased from 8.2 ± 1.2 to 3.5 ± 0.85, while in the laser group, pain levels dropped from 8.4 ± 1.4 to 2.6 ± 1.2. Additionally, shoulder pain and disability scores in the control group decreased from 102.9 ± 10.7 to 24 ± 10.3, and in the laser group, from 104.7 ± 11.8 to 22.6 ± 10.7 (P < 0.05). However, there was no significant difference between the two treatment groups in terms of neck and shoulder pain, disability, and range of motion (P > 0.05) (10). In contrast, our study observed a significant difference in these outcomes.

Alayat et al. found that after six weeks of treatment, MLS laser therapy combined with exercise resulted in significantly greater reductions in pain and disability scores compared to LLLT plus exercise (14), which aligns with our findings demonstrating the superior efficacy of MLS laser therapy. Additionally, Mehrdad et al. evaluated the effects of laser therapy on back pain and observed that laser therapy was more effective than exercise therapy in reducing pain and improving patient performance (13), further supporting our study results.

A review study by Hadizadeh et al. reported that three studies showed positive effects of low-power laser therapy in combination with basic treatments or as a standalone therapy for chronic and acute back pain (15). However, our study specifically demonstrated the effectiveness of MLS laser therapy in reducing pain in patients.

Morshidi et al. recommended laser therapy as a less invasive treatment method for reducing chronic lower spine pain (9). In our study, the effectiveness of MLS laser therapy for LBP was confirmed. Santamato et al. evaluated the impact of high-power laser therapy on pain severity in patients with chronic LBP and found that the laser-treated group experienced greater pain reduction and improved performance compared to the ultrasound group (16). Additionally, Vallone et al., while assessing the effects of diode laser therapy, reported pain reduction in both study groups; however, the improvement was more pronounced in the group that received laser therapy in combination with exercise (17).

In a randomized controlled study by Chen et al., it was stated that high-power laser therapy could increase the straight leg raise (SLR) angle and enhance overall patient performance (18). Similarly, Boyraz et al. compared the effects of high-power laser therapy and ultrasound in treating patients with lumbar discopathy and observed that exercise, high-power laser therapy, and ultrasound were all effective treatments (19). However, based on the present study and in comparison to other research in this field, laser therapy appears to significantly improve the functional performance of patients with chronic LBP.

### 5.1. Conclusions

According to the findings of this study and in comparison to previous research, MLS laser therapy can significantly reduce pain levels in patients with chronic LBP, with a greater reduction rate than that observed in the exercise group. Based on these results, MLS laser therapy may be a promising option for improving the condition of patients with chronic back pain. However, further studies with larger sample sizes are needed to validate these findings.

## Data Availability

The dataset presented in the study is available on request from the corresponding author during submission or after its publication. The data are not publicly available due to maintaining personal information of patients.

## Clinical Trial Registration Code:

IRCT20230827059275N1.
